# Management-associated herd-level risk factors for cryptosporidiosis in dairy calves in Finland

**DOI:** 10.3389/fpara.2026.1799503

**Published:** 2026-07-29

**Authors:** Tuulia Enbom, Leena Seppä-Lassila, Päivi J. Rajala-Schultz, Kristiina Sarjokari, Jenni Suolaniemi, Helena Rautala, Olli Ruoho, Sinikka Pelkonen, Tiina Autio

**Affiliations:** 1 Animal Health Diagnostic Unit, Finnish Food Authority, Kuopio, Finland; 2 Risk Assessment Unit, Finnish Food Authority, Helsinki, Finland; 3 Department of Production Animal Medicine, Faculty of Veterinary Medicine, University of Helsinki, Helsinki, Finland; 4 Valio Ltd., Helsinki, Finland; 5 Institute of Public Health and Clinical Nutrition, Faculty of Health Sciences, University of Eastern Finland, Kuopio, Finland; 6 Animal Health ETT, Seinäjoki, Finland

**Keywords:** diarrhea, calves, cryptosporidiosis, *Cryptosporidium parvum*, risk factors

## Abstract

**Introduction:**

Cryptosporidiosis is a prevalent diarrheal disease in young calves caused by the zoonotic protozoan parasite *Cryptosporidium parvum*. The etiology of the disease is multifactorial. This case–control study investigated herd-level risk factors associated with clinical cryptosporidiosis on dairy farms.

**Methods:**

The case farms (n=77) were Finnish dairy farms with clinical, laboratory-confirmed cryptosporidiosis and the control farms (n=152) were Finnish dairy farms without any recent clinical calf diarrhea outbreaks recorded in the most recent veterinarian's herd health visit. Control farms were not tested for the presence of *C. parvum*. Data were collected on overall herd health, management practices, environmental factors, and other factors potentially affecting the care of the calves. The data were analyzed with multivariable logistic regression.

**Results:**

The results indicate that a large herd size, cattle transfers between farms, insufficient isolation of sick calves from healthy ones, insufficient time for calf care, and five or more individuals involved in daily cattle work were all associated with increased risk of cryptosporidiosis.

**Discussion:**

The findings of this study suggest that several herd-level management factors were associated with the occurrence of cryptosporidiosis on dairy farms, and the external and internal biosecurity of farms should be improved. Information on potential factors predisposing to cryptosporidiosis can be used to target preventive management practices more effectively on cattle farms to prevent cryptosporidiosis in calves.

## 1 Introduction

Infectious diarrheal diseases are major illnesses of neonatal calves, and the most common pathogens are the protozoan parasite *Cryptosporidium parvum*, bovine rotavirus, bovine coronavirus, and the bacterium enterotoxigenic *Escherichia coli* F5 (K99) (ETEC) (Conrady et al., 2021). Calf diarrhea is a major cause of economic losses; it affects the welfare of calves and can predispose them to other diseases (Cho and Yoon, 2014; Calderon-Amor and Gallo, 2020). The main consequences on farms include reduced calf growth rates, increased mortality, higher treatment costs, and additional labor requirements.

*C. parvum* is a prevalent cause of diarrheal diseases in young calves, and cryptosporidiosis may cause problems, especially on dairy farms (Olson et al., 2004). Other common *Cryptosporidium* spp. species in cattle include *C. andersoni*, *C. bovis*, and *C. ryanae*, which are mainly associated with asymptomatic infections in older animals (Santin, 2020). The prevalence of *Cryptosporidium* is high in calves less than one month old in several countries (Smith et al., 2014; Maddox-Hyttel et al., 2006; Trotz-Williams et al., 2008). The route of infection is fecal–oral (Santin, 2020), and the infectious dose of *Cryptosporidium* is low; ingestion of as few as six protozoan oocysts is sufficient to cause infection (Zambriski et al., 2013). *C. parvum* is also a zoonotic pathogen and can cause serious intestinal infections in humans, which might require hospitalization and can even lead to death in the case of immunocompromised individuals (Kosek et al., 2001). Contact with cattle has been associated with *Cryptosporidium* infections in humans in many countries (Mcdaniel et al., 2014; Enbom et al., 2023; Roy et al., 2004). Farm workers and veterinarians have an occupational health risk of cryptosporidiosis as they may become infected while taking care of calves (Enbom et al., 2023). Therefore, the prevention of cryptosporidiosis on cattle farms is essential.

The emergence of clinical cryptosporidiosis is influenced by multiple host, pathogen, management, and environmental factors, including the calf’s immunological status, infection load, nutrition, management practices, and housing conditions (Trotz-Williams et al., 2007; Gulliksen et al., 2009). Several herd-level factors may predispose calves to *C. parvum* infection, but study results have been inconsistent (Delafosse et al., 2015; Brainard et al., 2020; Deksne et al., 2022). These risk factors include age of the calf, contacts with other calves, larger herds, organic production, various management practices, poor hygiene, the depth of the pen bedding, and other occurring pathogens. In a systematic review, Brainard et al. (2020) identified fourteen studies of which only three were considered methodologically strong and found no convincing evidence for any specific risk or protective factors. Therefore, more information on potential factors predisposing to cryptosporidiosis is needed to target management practices correctly to reduce the incidence of the disease.

*C. parvum* is a common endemic pathogen worldwide, and *Cryptosporidium* is prevalent in calves in many European countries (Maddox-Hyttel et al., 2006; Silverlås et al., 2009; Smith et al., 2014). In Finland, outbreaks in calves and humans were relatively rare until recently (Suominen et al., 2023; Finnish Food Authority, 2022). Data of laboratory-confirmed cryptosporidiosis from Finnish cattle herds show that the number of cryptosporidiosis cases has increased over fivefold in calves since 2012 (Finnish Food Authority, 2022). Since 2018, over 35% of examined herds whose samples were analyzed at the laboratory of the Finnish Food Authority have tested positive for *C. parvum* each year (The Zoonosis Centre, 2026). No previous studies in Finland have examined factors that may contribute to the increased occurrence of cryptosporidiosis in calves. The aim of our study was to identify factors associated with the clinical disease caused by *C. parvum* infection on dairy farms using a case–control study. This research paper presents information on management practices that are associated with occurrence of clinical cryptosporidiosis in calves on dairy farms, and may have implications to animal welfare and occupational hygiene of personnel working on farms.

## 2 Materials and methods

### 2.1 Study population and study design

This study was conducted among Finnish dairy herds belonging to the voluntary Centralized Cattle Health Care Register (Naseva register). In 2005, the Naseva register was developed in cooperation with Finnish dairy companies and slaughterhouses, and it is administered by Animal Health ETT, an industry-based association. The Naseva register has several interfaces to different databases, such as the Bovine Register, the Finnish Dairy Herd Recording System, databases of mastitis testing laboratories, meat inspection databases of slaughterhouses, and veterinary practice management systems. Herds in the Naseva must have a contract with a herd health veterinarian, who visits the farm regularly and has access to the farm’s production and medication data. The farmer and the farm’s veterinarian establish and implement a management plan that is updated during a specific herd health visit, which must be done at least once a year. This herd health visit includes monitoring of production data and animal movements, as well as observations of the condition, health, and behavior of different age groups of animals. Mortality in different age groups is also recorded in the database. Occurrence of signs and cases of bovine diseases, including diarrhea, is monitored and documented, and sampling is recommended when needed. The herd health veterinarian also monitors disease control measures, feed hygiene, and biosecurity. The evaluation performed by the veterinarian is documented in the Naseva register in a structured form using national predefined criteria categorized on a scale of 1, 2, or 3 (e.g., 1 = good practice, 2 = satisfactory practice, 3 = insufficient practice) or not applicable. National guidelines have been developed for farms to manage various diseases, including farm-level control of cryptosporidiosis. On January 31, 2019, 85% of Finnish dairy farms and 92% of cows were in the Naseva register.

**Figure 1:**
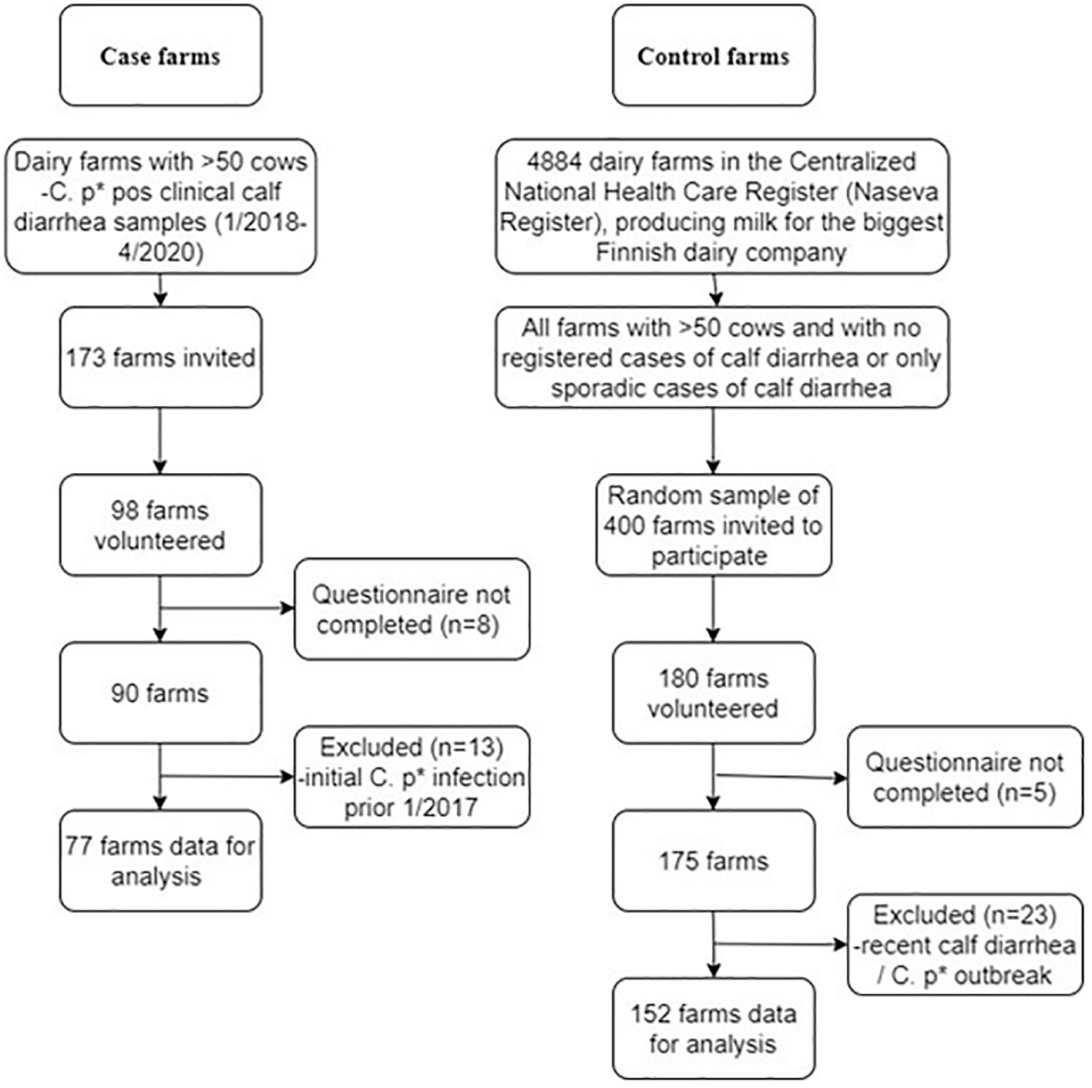
The inclusion and exclusion stages and criteria for case and control dairy herds in the study. *C.p: *Cryptosporidium parvum.*.

We conducted a case–control study to investigate potential risk factors for clinical cryptosporidiosis on farms. The study population comprised of Finnish dairy herds with at least 50 cows belonging to the Naseva register. The inclusion and exclusion criteria for case and control farms are presented in **Figure 1**.

A case farm was defined as a dairy farm with a calf diarrhea outbreak caused by laboratory confirmed *C. parvum* within the last two years. Farms with calf diarrhea outbreaks can send diagnostic samples from calves aged 0–5 weeks to the laboratory of the Finnish Food Authority for the detection of possible causative bacterial, parasitic or viral agents. Fecal samples were stained with the modified Ziehl–Neelsen stain for *Cryptosporidium* oocyst microscopy. The presence of *C. parvum* was confirmed by real time PCR. DNA was extracted using QIAamp Mini Kit (Qiagen, Hilden, German) according to the manufacturer’s protocol for tissue with overnight proteinase K treatment. The presence of *C. parvum* in samples was examined using real-time PCR and primers targeting the rRNA gene of *C. parvum* (CFX96 Touch Real-Time PCR Detection System, Bio-Rad Laboratories, CA, USA) as described previously (Burnet et al., 2013; Jothikumar et al., 2008). Commercially available plasmid pUC19 was used as an internal amplification control according to Fricker et al. (2007).

Farms that had a laboratory confirmed *C. parvum* calf diarrhea outbreak were contacted and invited to participate in this study.

A control farm was defined as a dairy farm without any calf diarrhea outbreaks including cryptosporidiosis outbreaks recorded in the most recent Naseva herd health visit (conducted within the past year). Status of calf health was checked from veterinarian’s annual health report from the Naseva register, and farms with *no calf diarrhea* or *only sporadic cases of calf diarrhea* were eligible to participate in the study. Control farms were not sampled for presence of *C. parvum*, because those farms had not had any calf diarrhea outbreaks. A total of 400 farms were randomly selected using the random number function in Excel and invited to participate in the study. To facilitate contact with the farms, the farms were selected within the biggest Finnish dairy company, which processes approximately 80% of milk produced in Finland.

The OpenEpi calculator for an unmatched case–control study (https://www.openepi.com/SampleSize/SSCC.htm) was used to determine the sample size to detect significant differences between case and control farms. The expected difference in the proportions of any management procedure or other feature between the case and control groups was set to 20% (40% vs. 60% in the calculations), and the control–case ratio was set at 2:1, the statistical power at 80%, and the confidence level at 95%. The calculated target sample size was 74 case farms and 147 control farms (and the least extreme odds ratio to be detected using this sample size was 2.25).

### 2.2 Data collection

Data on the study farms originated from several sources, and the participating farmers gave their informed consent to use their data in the study. The laboratory information management system of the Finnish Food Authority provided the information on *C. parvum* findings. Data on the number of animals on two separate dates with a 3-yr interval and on cattle transfers were obtained from the Bovine Register (Finnish Food Authority), herd health data from the Naseva register (ETT ry), and farm production data from the Finnish Dairy Herd Recording System operated by the Agricultural Data Processing Centre (ProAgria). **Table 1** summarizes the data and their sources.

**Table 1:** Description of the data used to evaluate management-associated risk factors for calf diarrhea caused by *Cryptosporidium parvum* in dairy calves.

Subject	Description	Type of variable	Source
Farm details	Herd size^1^ (≤60, 61 to 100, >100) Increase/decrease in herd size, number of animals^2^ (-40 to -11, -10 to 10, 11 to 40, 41 to 180)	Categorical Categorical	Bovine Register Bovine Register
	Herd average milk yield, kg^3^ Average parity^3^	Continuous Continuous	Finnish Dairy Herd Recording System
	Production type (organic/conventional)	Categorical	Naseva register
	Stall system (free stall barn, tie stall, other)	Categorical	Questionnaire^4^
Cattle transfers	No. of farms from which animals (all cattle, <1 mo calves specified) were purchased or otherwise transferred over a three-year period (0 to 1, 2 to 5, over 5)	Categorical	Bovine Register
Management practices			
Calving practices	Proportion of calves born in a calving pen (0%, 1-60%, 61% to 90%, 91% to 100%) Use of a calving pen for other purposes than calvings (a sick or treatment pen for cows, sick or temporary pen for calves or other or cannot say, no maternal pen) High calving peaks (no, a few high calving peaks, a considerable number of high calving peaks) Time a calf spends in a maternal pen (below 5 hours, over 5 hours, no maternal pen)	Categorical	Questionnaire^4^
Calf facilities	Pen type for unweaned calves (individual pens, pair pens, one group pen, two or more group pens, outdoor calf hutches) Transfer of calves from an individual pen or a pair pen (to a group pen with 3 to 10 calves, a group pen with 11 to 20 calves, an outdoor calf hutch or elsewhere, no information)	Categorical	Questionnaire^4^
	Facility index variable^5^	Categorical	Naseva register
Calf management practices	Age of unweaned calves in individual pens or pair pens (less than a week old, 1 to 2 weeks old, 3 to 4 weeks old, more than 4 weeks old, no individual or pair pens) Age difference between unweaned calves in a group pen (less than two weeks, 2 to 3 weeks, 4 to 5 weeks, 6 to 8 weeks, over 8 weeks, the situation varies) Period of time an individual pen or a pair pen is empty between unweaned calves (1 week or more, 3 to 6 days, 2 days or below, there are no individual or pair pens, I cannot say) Separation of sick calves from healthy calves (yes, always; yes, sometimes; no)	Categorical	Questionnaire^4^
Cattle management	No. of persons involved on a daily basis (1 to 2 persons, 3 to 4 persons, 5 persons or more) No. of persons involved during the past year (1 to 5 persons, 6 to 10 persons, over 10 persons)	Categorical	Questionnaire^4^
Calf management	Who usually takes care of calves (the farm owner/owners/spouse/other family member/substitute worker; employee(s) working with farm owner, spouse or other caretakers Adequacy of time in calf management (yes, always; yes, sometimes; no)	Categorical	Questionnaire^4^
Infectious diseases	Infectious disease epidemics in the herd during the past two years (yes, no) Diarrhea in adult cattle (yes, no) Diarrhea in calves (yes, no) Respiratory disease in adult cattle (yes, no) Respiratory disease in calves (yes, no) Interdigital phlegmon (yes, no)	Categorical	Questionnaire^4^
Disease prevention	Use of calf scour prophylaxis (yes, often/continuously; yes, sometimes; no)	Categorical	Questionnaire^4^
Visitors	Cattle transport personnel enter the calf facilities or group pen for calves (yes, always; yes, sometimes; no) Cattle transport personnel pick up calves from the barn door, from the outside or from a separate loading room (yes, always; yes, sometimes; no)	Categorical	Questionnaire^4^
Mental resources	Has the person responsible for taking care of calves been affected by any stressful event over the past year (yes, no)	Categorical	Questionnaire^4^

^1^ Number of cattle aged >24 mo at the time of the *C. parvum* outbreak (case farms) or survey completion (control farms).

^2^ Change in the number of cattle >24 mo of age in the three-year interval ending in the *C. parvum* outbreak (case farms) or survey (control farms).

^3^ During the year of the *C. parvum* outbreak (case farms) or year of the survey (control farms).

^4^ Information at the time of the *C. parvum* outbreak (case farms) or survey (control farms).

^5^ Water and feeding practices, general health, homogeneity of calf groups, air ventilation and temperature, space per animal, and bedding comfort evaluated by the herd health veterinarian in the Naseva register on a scale 1= good practice, 2= satisfactory practice, 3= insufficient practice.

Data on farm-level risk factors were collected with an online questionnaire with the Webropol 3.0 survey and reporting tool (Webropol Ltd, Helsinki, Finland) or via telephone. Telephone interview responses (n = 15) were entered into Webropol during the interviews. A structured questionnaire was developed to gather information about the workload of daily management, the disease status of the herd, and previous events on the farm that could have impacted, for example, the time spent on animal care. Information on calf management practices such as calving conditions, colostrum intake, and calf pens was also assessed. The questionnaire included a total of 51 questions. Content validation of the questionnaire was conducted through expert assessment of the clarity and relevance of the questions, complemented by pilot testing on five farms, two of which had experienced a *C. parvum* outbreak among calves. Subsequently, the questionnaire was modified based on the comments from the pilot respondents and experts, as well as observations made by the researchers. The questionnaire gathered data from the case farms concerning the time of the outbreak of *C. parvum* and from the control farms at the time of responding to the questionnaire. The data collection was conducted and the case and control farms completed the questionnaire between November 2019 and May 2020. Recall bias was possible because case farms answered with reference to the time when *C. parvum* was detected on the farm, whereas control farms responded according to the situation at the time of completing the questionnaire. The questionnaire is available on request in Finnish or Swedish from the first author.

### 2.3 Statistical analysis

We first calculated basic descriptive statistics on herd characteristics and the frequency of potential risk factors for cryptosporidiosis. We analyzed potential risk factors in calves using univariable analysis followed by multivariable logistic regression analysis. Variables with P < 0.2 in the univariable analysis were selected for inclusion in the multivariable logistic regression analysis. One commonly used method for reducing the number of predictor variables is to include in subsequent multivariable analyses only those variables that demonstrate a statistically significant unconditional association with the outcome in univariable screening, based on liberal significance criteria (e.g., *P* < 0.15 or *P* < 0.20) (Dohoo et al., 2014). The outcome of interest was a farm-level diagnosis of clinical cryptosporidiosis outbreak in calves (yes/no), in other words, a herd status as a case or a control farm. The associations between the outcome and potential farm-level risk factors were first assessed with univariable screening using the Mann–Whitney U-test for continuous variables or Fisher’s exact test for dichotomous and categorical variables with more than two levels.

Three variables with over five missing values were excluded from further analyses. The ​​responses were obtained from 77 case farms and 152 control farms. Thus, the total number of observations ​​was for 229 farms if the data for each farm were available. Variables with *P* < 0.2 in the univariable screening were assessed for collinearity with linear regression analysis using the variance inflation factor (VIF’) according to Shrestha (2020). No highly correlated variables were detected, as VIF was under 5 for every variable. Variables with *P* < 0.2 in the univariable analysis were first included in the multivariable logistic regression analysis simultaneously. Subsequently, a manual backward elimination of non-significant (*P* > 0.05) variables was performed with an examination of the results of the Wald test for individual variables, first dropping variables with the largest P-value, one at a time. Variables with *P* < 0.05 were considered significant and included in the final model. Biologically meaningful interactions were tested and kept in the final model if *P* < 0.05. No significant interactions were found. Statistical analyses were performed using IBM^®^ SPSS Statistics software Version 27 (27.0.1.0) (IBM Corp. Released 2020. IBM SPSS Statistics for Windows, Version 27.0. Armonk, NY: IBM Corp).

## 3 Results

### 3.1 Descriptive statistics and risk factors

Altogether, 90 case and 175 control farms completed the questionnaire: the response rates were 52% (90/173) and 44% (175/400), respectively. From the analysis, 13 case farms were excluded because *C. parvum* had been diagnosed for the first time on the farm prior to 2017 and the survey was conducted between November 2019 and May 2020. This exclusion was made because the respondents would not have necessarily remembered the events at the time when *C. parvum* was first detected on the farm. Based on the inclusion and exclusion criteria in **Figure 1**, a total of 77 case farms and 152 control farms were included in the final analysis.

The median herd size was 93 cows on the case farms and 69 cows on the control farms (**Table 2**). Cattle transfers were more common on the case farms than on the control farms. During the three-year study period, the number of animals had increased considerably, by 41–180 cows, on 16.9% (13/77) of the case farms. On the control farms, only 5.3% (8/152) had a corresponding increase in the number of animals. On 69.7% of the control farms, the number of animals had remained relatively stable, increasing or decreasing by a maximum of 10 cows, and the corresponding proportion of case farms was 59.7%.

**Table 2:** Basic descriptive data for case and control farms, using Fisher’s exact test for categorical variables and the Mann–Whitney U-test for continuous variables when comparing case and control farms.

Variable	Case farms (n = 77)	Control farms (n = 152)	*P*-value	
	n/n_tot_ (%)	n/n_tot_ (%)		
Barn type				
Free stall	72/77 (93.5)	134/152 (88.2)	0.249	
Tie stall	9/77 (11.7)	24/152 (15.8)	0.435	
Other stall system	0/77 (0.0)	3/152 (2.0)	0.553	
Organic production	2/77 (2.6)	4/152 (2.6)	1.000	
Herd size^1^			<0.001	
≤60	11/77 (14.3)	45/152 (29.6)		
61 to 100	31/77 (40.3)	82/152 (53.9)		
>100	35/77 (45.5)	25/152 (16.4)		
Change in the number of cattle^2^			0.047	
-10 to 10	46/77 (59.7)	106/152 (69.7)		
-40 to -11	4/77 (5.2)	9/152 (5.9)		
11 to 40	14/77 (18.2)	29/152 (19.1)		
41 to 180	13/77 (16.9)	8/152 (5.3)		
Cattle transfers^3^			0.006	
0 to 1	40/77 (51.9)	108/152 (71.1)		
2 to 5	24/77 (31.2)	35/152 (23.0)		
Over 5	13/77 (16.9)	9/152 (5.9)		
<1-mo-old calf transfers^3^			0.447	
0 to 1	73/77 (94.8)	148/152 (97.4)		
2 to 5	4/77 (5.2)	4/152 (2.6)		
	Median (sd)	Median (sd)		National database 2019^4^
Herd size^1^	93 (49)	69 (27)	<0.001	48
Average milk yield (kg/cow)^5^	10 103 (906)	10 143 (1188)	0.777	9937
Average parity^5^	2.5 (0.3)	2.7 (0.4)	0.007	2.57

^1^ Number of cattle >24 mo of age at the time of the *Cryptosporidium parvum* outbreak (case farms) or survey (control farms).

^2^ Change in the number of cattle >24 mo of age in the three-year interval ending in the *C. parvum* outbreak (case farms) or survey (control farms).

^3^ No. of farms from which animals were purchased or obtained over a three-year period.

^4^ Results of dairy cattle production monitoring in 2019 from the Finnish Dairy Herd Recording System (ProAgria).

^5^ Missing data: 2 case farms, 10 control farms.

In the initial univariable screening, several variables were associated with clinical cryptosporidiosis (**Table 2**, **3**). Results of the multivariable logistic regression analysis (**Table 4**) were obtained from 76 case farms and 150 control farms; one case farm and two control farms had some missing data and were thus omitted from the analysis. These results demonstrated that a herd size of more than 100 animals over 24 months of age (OR 3.4), cattle transfers from two or more herds (OR 3.0) or more than five transfers (OR 7.7), insufficient time for calf care (OR 45.2), and five or more individuals involved in daily cattle work (OR 8.3) increased the odds of being a case farm. Although not separating sick calves from healthy ones was not significantly associated with case farm status (adjusted OR = 3.1, 95% CI 0.97–9.83; *P* = 0.056) compared to always separating them, the practice still indicated a tendency toward clearly higher odds of being a case farm. Diarrhea in adult cattle in the herd reported by a farmer, more resources (6–10 or more than 10 persons) in cattle work during the preceding year, and if a respondent had experienced a stressful event over the previous year were observed to be associated with lower odds of being a case farm. The logistic regression model showed relatively strong explanatory power, with a Nagelkerke R² value of 0.46. The Hosmer–Lemeshow test yielded a P−value of 0.294, which is not statistically significant (P > 0.05), indicating that the model demonstrates good fit.

**Table 3:** Descriptive data on risk factors for cryptosporidiosis on case and control farms.

	Case farms	Control farms	
Variable	Yes (n/n, %)	Yes (n/n, %)	*P*-value
Diarrhea in adult cattle	14/77, 18.2	57/151, 37.7	0.003
Respiratory disease in adult cattle	7/77, 9.1	27/151, 17.9	0.114
Does the farm use calf scour prophylaxis in cows and heifers because of calf diarrhea?			0.003
Yes, often/continuously	12/77, 15.6	6/152, 3.9	
Yes, sometimes	5/77, 6.5	5/152, 3.3	
No	60/77, 77.9	141/152, 92.8	
Separation of sick calves from healthy calves			0.010
Yes, always	27/77, 35.1	54/152, 35.5	
Yes, sometimes	34/77, 44.2	87/152, 57.2	
No	16/77, 20.8	11/152, 7.2	
High calving peaks on the farm in the last year			0.023
No	9/77, 11.7	19/152, 12.5	
A few high calving peaks	49/77, 63.6	117/152, 77.0	
A considerable number of high calving peaks	19/77, 24.7	16/152, 10.5	
The time a calf spends in a maternal pen			0.162
Less than ​5 hours	41/77, 53.2	64/152, 42.1	
Over 5 hours	26/77, 33.8	71/152, 46.7	
No maternal pen	10/77, 13.0	17/152, 11.2	
Individual pens are used for young calves	57/77, 74.0	120/152, 78.9	0.408
Pair pens are used for young calves	7/77, 9.1	25/152, 16.4	0.159
There is one group pen for young calves	18/77, 23.4	30/152, 19.7	0.606
There are two or more group pens for young calves	42/77, 54.5	108/152, 71.1	0.018
Outdoor calf hutches were used for young calves	14/77, 18.2	12/152, 7.9	0.027
The time for which the individual pen or the pair pen was empty before the new calf was transferred to the pen			0.134
1 week or more	9/76, 11.8	40/152, 26.3	
3 to 6 days	22/76, 28.9	36/152, 23.7	
2 days or less	26/76, 34.2	43/152, 28.3	
No individual or pair pens in use on the farm	13/76, 17.1	20/152, 13.2	
I cannot say	6/76, 7.9	13/152, 8.6	
The person taking the calves to the rearing farm visited the group pen for calves			0.110
Yes, always	0/74, 0.0	1/150, 0.7	
Yes, sometimes	7/74, 9.5	5/150, 3.3	
No	67/74, 90.5	144/150, 96.0	
The person taking the calves to the rearing farm picked up the calves from the barn door, from the outside or from a separate loading room			0.027
Yes, always	60/75, 80.0	138/151, 91.4	
Yes, sometimes	9/75, 12.0	5/151, 3.3	
No	6/75, 8.0	7/151, 4.6	
I cannot say	0/75, 0.0	1/151, 0.7	
The calves are cared for			0.002
by the farm owner/owners/spouse/other family member/substitute worker	38/77, 49.4	107/151, 70.9	
by employee(s) working with aforementioned caretakers	39/77, 50.6	44/151, 29.1	
The number of persons involved in livestock work on a daily basis under normal circumstances			0.039
1 to 2 persons	44/77, 57.1	109/151, 72.2	
3 to 4 persons	30/77, 39.0	40/151, 26.5	
5 persons or more	3/77, 3.9	2/151, 1.3	
The number of persons involved in taking care of cattle during the last year			0.105
1 to 5 persons	36/77, 46.8	50/152, 32.9	
6 to 10 persons	31/77, 40.3	72/152, 47.4	
Over 10 persons	10/77, 13.0	30/152, 19.7	
Adequacy of the time allocated to calf care			<0.001
Yes, always enough time	43/77, 55.8	116/152, 76.3	
Yes, sometimes enough time	22/77, 28.6	34/152, 22.4	
No, not enough time for calf care	12/77, 15.6	2/152, 1.3	
A stressful event during the past year	28/76, 36.8	78/152, 51.3	0.049
Facility index variable			0.049
Good practice	62/76, 81.6	140/152, 92.1	
Satisfactory practice	12/76, 15.8	11/152, 7.2	
Insufficient practice	2/76, 2.6	1/152, 0.7	

*P*-values are from univariable analysis comparing case and control farms using Fisher’s exact test.

**Table 4:** Results from a multivariable logistic regression model for the odds of being a case farm with cryptosporidiosis outbreak in calves.

Factor	β	Adjusted OR (95% CI)	*P*-value
Herd size^1^			0.007*
≤60	ref.		
61 to 100	-0.10	0.91 (0.34 to 2.43)	0.848
>100	1.23	3.41 (1.16 to 10.04)	0.026
Cattle transfers^2^			0.003*
0 to 1	ref.		
2 to 5	1.11	3.04 (1.21 to 7.68)	0.018
Over 5	2.04	7.72 (2.19 to 27.16)	0.001
Separation of sick calves from healthy calves			0.006*
Yes, always	ref.		
Yes, sometimes	-0.68	0.51 (0.23 to 1.12)	0.092
No	1.13	3.09 (0.97 to 9.83)	0.056
Adequacy of the time allocated to calf care			<0.001*
Yes, always enough time	ref.		
Yes, sometimes enough time	1.38	3.96 (1.57 to 9.99)	0.003
No, not enough time for calf care	3.81	45.15 (6.51 to 313.11)	<0.001*
The number of persons involved in daily cattle work under normal circumstances			0.028
1 to 2 persons	ref.		
3 to 4 persons	0.95	2.59 (1.16 to 5.80)	0.021
5 persons or more	2.12	8.29 (0.87 to 78.73)	0.066
The number of persons involved in taking care of cattle during the last year			0.019
1 to 5 persons	ref.		
6 to 10 persons	-0.82	0.44 (0.19 to 1.01)	0.053
Over 10 persons	-1.63	0.20 (0.06 to 0.65)	0.008
Diarrhea in adult cattle: no	ref.		
Yes	-1.25	0.29 (0.12 to 0.68)	0.005
A stressful event during the past year^3^: no	ref.		
Yes	-1.45	0.24 (0.09 to 0.59)	0.002
Constant	0.50		0.196

The results include 76 case farms and 150 control farms.

β = estimated coefficient.

OR = odds ratio.

CI = confidence interval.

^1^ No. of cattle aged >24 mo.

^2^ No. of farms from which animals were purchased or obtained over a three-year period.

^3^ Has the person responsible for taking care of calves been affected by any stressful event over the past year.

ref. = reference.

* = *P* < 0.05.

## 4 Discussion

The study identified several factors associated with farms with clinical cryptosporidiosis in calves. These were a large herd size, more cattle transfers between farms, no separation of sick calves from healthy ones, many individuals involved in daily cattle work, and insufficient time for calf care. The odds of being a case farm decreased with increasing number of persons involved in cattle work during the preceding year. The odds were also lower if adult cattle in the farm had had diarrhea and if the respondents had experienced a stressful event during the previous year compared to a situation if they had not. However, these findings may reflect reporting or time frame differences, or residual confounding rather than true protective effects. Notably, data from the case farms were collected in relation to the time of the outbreak, whereas control farms reported the situation at the time of the survey. This difference in the time frame used for data collection may have affected the comparability of several management-related variables, such as time allocated to calf care, the respondent’s experience of a stressful event, diarrhea in adult cattle, and labor availability.

The control farms were not tested for *C. parvum* and therefore, they may have included farms with subclinical or unrecognized infections. Consequently, the findings of this study should be interpreted as factors associated with clinical, laboratory-confirmed outbreaks on dairy farms rather than with *C. parvum* infection more broadly. The inclusion criterion for farms in this study was a herd size of more than 50 dairy cows, indicating that the participating farms were slightly larger than the average Finnish dairy farm, as shown in the 2019 dairy production monitoring data from the Finnish Dairy Herd Recording System (**Table 2**). Nevertheless, the farms constitute a representative cross-section of the Finnish dairy population, and the results are likely generalizable to farms beyond the study population.

The finding that a herd size of more than 100 cows was associated with the clinical cryptosporidiosis compared to a herd size of less than 100 cows is consistent with previous reports. A herd size of more than 100 adult animals (Duranti et al., 2009), 500 cows (Urie et al., 2018), or 200 animals including all ages (Szonyi et al., 2012) has been found to increase the risk of *C. parvum* infection. Larger herds have a larger number of susceptible young calves, which may become infected with *C. parvum*, increasing the environmental contamination of the calf premises (Urie et al., 2018; Duranti et al., 2009) and leading to a chain of infection. Furthermore, management factors, which likely vary with herd size, are associated with the prevalence of *Cryptosporidium* (Urie et al., 2018). A large herd size is likely to be associated with many people involved in daily cattle work. Based on the univariable analyses, we also found that the number of cows had increased considerably during the three-year study period on many of the case farms. This is likely due to animal additions. Bringing new animals onto the farm is a biosecurity concern and may pose a risk of introducing more diseases to the farm. Many factors might influence the association between herd size and animal health and welfare, such as herd expansion, facilities, management, the training and experience of farm workers, and the ratio of workers to the number of animals (Barkema et al., 2015).

Cattle transfers were associated with the occurrence of clinical cryptosporidiosis. Expanding the herd by purchasing cattle, sending heifer calves to be raised on another farm, and reintroducing the farm’s own cattle upon returning, for example, from another farm, agricultural shows, common pastures, or contract rearing facilities are potential routes for the transfer of infectious diseases from one farm to another, and hence create a high biosecurity risk (Mee et al., 2012). However, import of cattle to Finland from other countries is nearly non-existent, contacts between animals from different farms are limited as common pastures and cattle shows are rare, and public livestock markets and auctions are non-existent (Autio et al., 2021). External biosecurity includes all measures to prevent pathogen entry to the farm, and an effective way to prevent the risk of infection is to maintain a closed herd without introducing new animals (Mee et al., 2012; Damiaans et al., 2020).

Even though our study did not demonstrate unambiguous significant associations between case farm status and separation of sick calves from healthy ones, this practice can be an important management procedure in preventing the spread of diseases with a herd. Internal farm biosecurity includes measures to prevent the spread of pathogens within the farm, and the separation of sick animals from healthy ones is crucial to prevent the spread of infection (Damiaans et al., 2020). In addition, hygiene measures, such as a dry and clean environment, are generally important components in disease control (Santin, 2020). The control of cryptosporidiosis is difficult, because oocysts are resistant to many disinfectants (Delling et al., 2017) and are well preserved, especially in humid conditions (Ryan et al., 2014). A calf can obtain oocysts from a contaminated pen, dirty water and feeding containers, or by licking other calves (Delafosse et al., 2015) or human clothing contaminated with manure (Smith et al., 2014). Silverlås et al. (2009) observed a higher prevalence of *Cryptosporidium* spp. in dairy herds using a continuous or a mixed system for moving young stock compared to an all-in/all-out system. This suggests that the spread of *Cryptosporidium* can at least partially be controlled by barriers. Hospital pens for diseased animals should be separate from healthy animals to prevent disease transmission (Gorden and Plummer, 2010), and it is advisable to have hospital pens or individual hutches in a separate area for sick calves. Additionally, efficient cleaning and disinfection procedures can help limit the spread of diseases on a farm. Investments in the design of calf premises should be made as a part of barn design to make farm solutions functional for disease control, considering that enough pens for calves exist, calf facilities are easy to clean, and the pen structures can be dried.

In the current study, farmer’s perceptions regarding inadequate time being allocated to caring for calves was also associated with case farm status. Therefore, sufficient labor and time should be reserved for calf care. However, it is important to note that the confidence intervals for the ‘no, not enough time for calf care’ category were wide, indicating substantial uncertainty around the estimate. Interestingly, 5 or more individuals being involved in daily cattle work compared to 1–2 people caring for cattle daily was associated with an increased odds of clinical cryptosporidiosis, albeit association was not statistically significant. However, having a greater number of different people (e.g., family members, substitute workers, trainees) working on a farm during the previous year, was associated with decreased odds of being a case farm. This may reflect the fact that the control farms reported more labor compared to the case farms, which may have reduced the workload per person on the farm. A higher proportion of respondents from the control than from the case farms reported that they had experienced some stressful event in the past year. This might have affected their use of time or resources. The questionnaire did not specify details about which stressful event(s) the respondent had experienced.

Diarrhea in adult cattle was negatively associated with the odds of being a case farm in our multivariable model. The control farms reported significantly more often diarrhea in adult cattle compared to the case farms. This was most likely related to the time of the survey, since epidemics of bovine coronavirus, both winter dysentery and respiratory disease, were commonly observed throughout Finland in the winter of 2019–2020 (Finnish Food Authority, 2022). That was the time when the control farms responded to the questionnaire. The case farms, instead, responded to the survey concerning the time when cryptosporidiosis had emerged in their herd, and for a large number of them it was before the winter of 2019–2020. Maybe winter dysentery had not occurred during the time period that the questionnaire concerned, or the respondents had forgotten about the diarrhea cases in adult cattle. Another possible hypothesis is that the control farms may be better at recognizing and treating sick animals, which could contribute to their greater effectiveness in limiting the spread of cryptosporidiosis among calves.

## 5 Limitations of the study

Recall bias is possible in case–control studies (Coughlin, 1990). In this study, this bias is possible because the case farms responded to the questionnaire regarding the situation at the time of the *C. parvum* outbreak whereas the control farms answered according to the situation at the time of the survey. According to the survey results, the respondents from the control farms reported that they had experienced some stressful events more frequently than those on the case farms. The difference in timing may have affected how well the respondents remembered stressful events and may have caused some recall bias. The responses relating to different times from the case and control farms probably also influenced the fact that diarrhea in adult cattle was found as a protective factor in the study. Ideally, the time frame for answering the questionnaire would have been consistent for both case and control farms, but in order to include a sufficient number of case farms, the retrospective time period had to be longer. The control group was asked to answer the questionnaire based on the situation at the time of responding and on events from the previous year in order to decrease recall bias. Thirteen case farms in which cryptosporidiosis had been diagnosed prior to 2017 were excluded from the study to prevent substantial differential timing of exposure assessment between cases and controls from affecting the results.

Due to voluntary participation in the survey, the possibility of selection bias cannot be excluded, which may limit the generalizability of the results to the entire Finnish cattle population. Nevertheless, we believe that our study population was quite representative of the Finnish dairy herds at the time of the study. The average milk yield in the study herds was similar to that in Finnish dairy population in general and it was very similar between case and control farms. The case farms, however, were larger and had a lower average parity than the control farms. Voluntary participation may have affected this.

Non-response bias is possible due to voluntary participation in the study, with the questionnaire response rate being 52% for the case farms and 44% for the control farms. Information bias and social desirability bias may also have occurred because farmers self-reported their responses by answering the questionnaire. Several variables, including the adequacy of time allocated to calf care, were based on farmers’ subjective assessments and may therefore represent imperfect or heterogeneous measures across farms. As evaluations of time use are inherently subjective, individual differences in perception, recall, and interpretation may have resulted in inconsistency in how respondents assessed the time demands of calf care.

Data of herd health visits documented in the Naseva register are based on the evaluations made by numerous herd health veterinarians. Therefore, there may be some variation in how different veterinarians evaluate farms against the national pre-defined criteria. However, this potential bias is likely to influence the data of both case and control farms in a similar way. Such non-differential information bias typically biases the results toward the null, thereby reducing the apparent effects of the factors under this study.

## 6 Conclusion

Cryptosporidiosis caused by *C. parvum* in calves is associated with a larger herd size, and the risk factors are management-related. Careful attention should be paid to external and internal biosecurity of dairy farms. Hospital pens in the barn indicate a potential role in reducing transmission of the infection by allowing separation of sick and healthy calves. Caring for calves requires labor and time, and the occurrence of cryptosporidiosis may be reduced by investing in calf care and risk management. Investing in calf management and health, as well as controlling factors associated with cryptosporidiosis in calves, may help reduce human cases.

## Data Availability

The farmers provided consent for the use of their data only for the purposes of this study. Any further use of the data requires separate permission. Requests regarding data access or reuse should be directed to the corresponding author..

## References

[B1] Autio T. Tuunainen E. Nauholz H. Pirkkalainen H. London L. Pelkonen S. (2021). Overview of control programs for cattle diseases in Finland. Front. Vet. Sci. 8. doi: 10.3389/fvets.2021.688936 34395573 PMC8361753

[B2] Barkema H. W. Von Keyserlingk M. A. Kastelic J. P. Lam T. J. Luby C. Roy J. P. . (2015). Invited review: Changes in the dairy industry affecting dairy cattle health and welfare. J. Dairy Sci. 98, 7426–7445. doi: 10.3168/jds.2015-9377 26342982

[B3] Brainard J. Hooper L. Mcfarlane S. Hammer C. C. Hunter P. R. Tyler K. (2020). Systematic review of modifiable risk factors shows little evidential support for most current practices in Cryptosporidium management in bovine calves. Parasitol. Res. (1987) 119, 3571–3584. doi: 10.1007/s00436-020-06890-2 PMC7524573 32996051

[B4] Burnet J. B. Ogorzaly L. Tissier A. Penny C. Cauchie H. M. (2013). Novel quantitative TaqMan real‐time PCR assays for detection of Cryptosporidium at the genus level and genotyping of major human and cattle‐infecting species. J. Appl. Microbiol. 114, 1211–1222. doi: 10.1111/jam.12103 23230846

[B5] Calderon-Amor J. Gallo C. (2020). Dairy calf welfare and factors associated with diarrhea and respiratory disease among Chilean dairy farms. Anim. (Basel) 10, 1115. doi: 10.3390/ani10071115 32610569 PMC7401522

[B6] Cho Y.-I. Yoon K.-J. (2014). An overview of calf diarrhea - infectious etiology, diagnosis, and intervention. J. Veterinary Sci. (Suwon-si Korea) 15, 1–17. doi: 10.4142/jvs.2014.15.1.1 24378583 PMC3973752

[B7] Conrady B. Brunauer M. Roch F. F. (2021). Cryptosporidium spp. infections in combination with other enteric pathogens in the global calf population. Anim. (Basel) 11, 1786. doi: 10.3390/ani11061786 34203818 PMC8232747

[B8] Coughlin S. S. (1990). Recall bias in epidemiologic studies. J. Clin. Epidemiol. 43, 87–91. doi: 10.1016/0895-4356(90)90060-3 2319285

[B9] Damiaans B. Renault V. Sarrazin S. Berge A. C. Pardon B. Saegerman C. . (2020). A risk-based scoring system to quantify biosecurity in cattle production. Prev. Veterinary Med. 179, 104992–104992. doi: 10.1016/j.prevetmed.2020.104992 32438203

[B10] Deksne G. Mateusa M. Cvetkova S. Derbakova A. Keidāne D. Troell K. . (2022). Prevalence, risk factor and diversity of Cryptosporidium in cattle in Latvia. Veterinary Parasitol. 28, 100677–100677. doi: 10.1016/j.vprsr.2021.100677 35115117

[B11] Delafosse A. Chartier C. Dupuy M. C. Dumoulin M. Pors I. Paraud C. (2015). Cryptosporidium parvum infection and associated risk factors in dairy calves in western France. Prev. Veterinary Med. 118, 406–412. doi: 10.1016/j.prevetmed.2015.01.005 PMC7172863 25623968

[B12] Delling C. Lendner M. Muller U. Daugschies A. (2017). Improvement of *in vitro* evaluation of chemical disinfectants for efficacy on Cryptosporidium parvum oocysts. Vet. Parasitol. 245, 5–13. doi: 10.1016/j.vetpar.2017.07.018 28969838

[B13] Dohoo I. Martin W. Stryhn H. (2014). Veterinary Epidemiologic Research. 2nd ed (Charlottetown, Prince Edward Island: VER Inc).

[B14] Duranti A. Cacciò S. M. Pozio E. Di Egidio A. De Curtis M. Battisti A. . (2009). Risk factors associated with Cryptosporidium parvum infection in cattle. Zoonoses Public Health 56, 176–182. doi: 10.1111/j.1863-2378.2008.01173.x 18771517

[B15] Enbom T. Suominen K. Laitinen S. Ollgren J. Autio T. Rimhanen-Finne R. (2023). Cryptosporidium parvum: an emerging occupational zoonosis in Finland. Acta Vet. Scand. 65, 25–25. doi: 10.1186/s13028-023-00684-z 37349848 PMC10286481

[B16] ETT ry Naseva Register. Centralized Cattle Health Care Register. Available online at: https://www.naseva.fi/PublicContent/IntroductionInEnglish (Accessed March 10, 2024).

[B17] Finnish Food Authority Bovine Register. Available online at: https://www.mtech.fi (Accessed March 10, 2024).

[B18] Finnish Food Authority (2022). Animal Diseases in Finland 2021. Available online at: https://www.ruokavirasto.fi/globalassets/tietoa-meista/julkaisut/julkaisusarjat/julkaisuja/elaimet/animal-diseases-in-Finland-2021.pdf (Accessed May 15, 2026).

[B19] Fricker M. Messelhausser U. Busch U. Scherer S. Ehling-Schulz M. (2007). Diagnostic real-time PCR assays for the detection of emetic Bacillus cereus strains in foods and recent food-borne outbreaks. Appl. Environ. Microbiol. 73, 1892–1898. doi: 10.1128/aem.02219-06 17259359 PMC1828801

[B20] Gorden P. J. Plummer P. (2010). Control, management, and prevention of bovine respiratory disease in dairy calves and cows. Veterinary Clinics North America Food. Anim. Pract. 26, 243–259. doi: 10.1016/j.cvfa.2010.03.004 20619182 PMC7135383

[B21] Gulliksen S. M. Jor E. Lie K. I. Hamnes I. S. Løken T. Åkerstedt J. . (2009). Enteropathogens and risk factors for diarrhea in Norwegian dairy calves. J. Dairy Sci. 92, 5057–5066. doi: 10.3168/jds.2009-2080 19762824 PMC7094401

[B22] Jothikumar N. da Silva A. J. Moura I. Qvarnstrom Y. Hill V. R. (2008). Detection and differentiation of Cryptosporidium hominis and Cryptosporidium parvum by dual TaqMan assays. J. Med. Microbiol. 57, 1099–1105. doi: 10.1099/jmm.0.2008/001461-0 18719179

[B23] Kosek M. Alcantara C. Lima A. M. Guerrant R. L. (2001). Cryptosporidiosis: an update. Lancet Infect. Dis. 1, 262–269. doi: 10.1016/s1473-3099(01)00121-9 11871513

[B24] Maddox-Hyttel C. Langkjær R. B. Enemark H. L. Vigre H. (2006). Cryptosporidium and Giardia in different age groups of Danish cattle and pigs—Occurrence and management associated risk factors. Veterinary Parasitol. 141, 48–59. doi: 10.1016/j.vetpar.2006.04.032 16797848

[B25] Mcdaniel C. J. Cardwell D. M. Moeller R. B. Gray G. C. (2014). Humans and cattle: a review of bovine zoonoses. Vector Borne Zoonotic Dis. (Larchmont N.Y.) 14, 1–19. doi: 10.1089/vbz.2012.1164 24341911 PMC3880910

[B26] Mee J. F. Geraghty T. O’neill R. More S. J. (2012). Bioexclusion of diseases from dairy and beef farms: risks of introducing infectious agents and risk reduction strategies. Veterinary J. (1997) 194, 143–150. doi: 10.1016/j.tvjl.2012.07.001 PMC7110757 23103219

[B27] Olson M. E. O'handley R. M. Ralston B. J. Mcallister T. A. Andrew Thompson R. C. (2004). Update on Cryptosporidium and Giardia infections in cattle. Trends Parasitol. 20, 185–191. doi: 10.1016/j.pt.2004.01.015 15099558

[B28] ProAgria Finnish Dairy Herds Recording System. Available online at: https://www.proagria.fi/en (Accessed March 10, 2024).

[B29] Roy S. L. Delong S. M. Vugia D. J. Zansky S. M. Dietz V. Beach M. J. . (2004). Risk factors for sporadic cryptosporidiosis among immunocompetent persons in the United States from 1999 to 2001. J. Clin. Microbiol. 42, 2944–2951. doi: 10.1128/jcm.42.7.2944-2951.2004 15243043 PMC446318

[B30] Ryan U. N. A. Fayer R. Xiao L. (2014). Cryptosporidium species in humans and animals: current understanding and research needs. Parasitology 141, 1667–1685. doi: 10.1017/s0031182014001085 25111501

[B31] Santin M. (2020). Cryptosporidium and giardia in ruminants. Veterinary Clinics North America Food. Anim. Pract. 36, 223–238. doi: 10.1016/j.cvfa.2019.11.005 32029186

[B32] Shrestha N. (2020). Detecting multicollinearity in regression analysis. Am. J. Appl. Mathematics Stat 8, 39–42. doi: 10.12691/ajams-8-2-1

[B33] Silverlås C. Emanuelson U. De Verdier K. Björkman C. (2009). Prevalence and associated management factors of Cryptosporidium shedding in 50 Swedish dairy herds. Prev. Veterinary Med. 90, 242–253. doi: 10.1016/j.prevetmed.2009.04.006 19443061

[B34] Smith R. P. Clifton-Hadley F. A. Cheney T. Giles M. (2014). Prevalence and molecular typing of Cryptosporidium in dairy cattle in England and Wales and examination of potential on-farm transmission routes. Veterinary Parasitol. 204, 111–119. doi: 10.1016/j.vetpar.2014.05.022 PMC7115801 24909077

[B35] Suominen K. A. Björkstrand M. Ollgren J. Autio T. J. Rimhanen-Finne R. (2023). Cryptosporidiosis in Finland is predominantly of domestic origin: investigation of increased reporting 1995–2020. Infect. Dis. 55, 116–124. doi: 10.1080/23744235.2022.2136749 36266958

[B36] Szonyi B. Chang Y.-F. Wade S. E. Mohammed H. O. (2012). Evaluation of factors associated with the risk of infection with Cryptosporidium parvum in dairy calves. Am. J. Veterinary Res. 73, 76–85. doi: 10.2460/ajvr.73.1.76 22204291

[B37] The Zoonosis Centre (2026). Zoonoses in Finland in 2005-2024. Available online at: https://www.ruokavirasto.fi/globalassets/teemat/zoonoosikeskus/zoonoosit/loisten-aiheuttamat-taudit/3_2_krypto_elaimet.pdf (Accessed June 8, 2026).

[B38] Trotz-Williams L. A. Martin S. W. Leslie K. E. Duffield T. Nydam D. V. Peregrine A. S. (2008). Association between management practices and within-herd prevalence of Cryptosporidium parvum shedding on dairy farms in southern Ontario. Prev. Veterinary Med. 83, 11–23. doi: 10.1016/j.prevetmed.2007.03.001 17481752 PMC7114088

[B39] Trotz-Williams L. A. Wayne Martin S. Leslie K. E. Duffield T. Nydam D. V. Peregrine A. S. (2007). Calf-level risk factors for neonatal diarrhea and shedding of Cryptosporidium parvum in Ontario dairy calves. Prev. Veterinary Med. 82, 12–28. doi: 10.1016/j.prevetmed.2007.05.003 17602767 PMC7114353

[B40] Urie N. J. Lombard J. E. Shivley C. B. Adams A. E. Kopral C. A. Santin M. (2018). Preweaned heifer management on US dairy operations: Part III. Factors associated with Cryptosporidium and Giardia in preweaned dairy heifer calves. J. Dairy Sci. 101, 9199–9213. doi: 10.3168/jds.2017-14060 29859689

[B41] Zambriski J. A. Nydam D. V. Wilcox Z. J. Bowman D. D. Mohammed H. O. Liotta J. L. (2013). Cryptosporidium parvum: determination of ID50 and the dose–response relationship in experimentally challenged dairy calves. Veterinary Parasitol. 197, 104–112. doi: 10.1016/j.vetpar.2013.04.022 23680540 PMC7116995

